# Rethinking preparedness planning in disaster emergency care: lessons from a beyond-surge-capacity event

**DOI:** 10.1186/s13017-021-00403-x

**Published:** 2021-11-29

**Authors:** Sheuwen Chuang, David D. Woods, Morgan Reynolds, Hsien-Wei Ting, Asher Balkin, Chin-Wang Hsu

**Affiliations:** 1 Graduate Institute of Data Science, Taipei Medical University, Taipei, Taiwan; 2 TMU Research Center of Health and Welfare Policy, Taipei Medical University, 12F, No. 172-1, Sec. 2 Keelung Rd. Da an Dist., Taipei City, Taiwan; 3 Department of Integrated Systems Engineering, The Ohio State University, Columbus, OH US; 4 Department of Neurosurgery, Taipei Hospital, Ministry of Health and Welfare, New Taipei City, Taiwan; 5 Emergency Department, Taipei Municipal Wanfang Hospital, Taipei, Taiwan

**Keywords:** Mass casualty incident, Formosa Fun Coast Dust Explosion, Preparedness plan, Emergency medical service, Surge capacity, Burn disaster

## Abstract

**Background:**

Large-scale burn disasters can produce casualties that threaten medical care systems. This study proposes a new approach for developing hospital readiness and preparedness plan for these challenging beyond-surge-capacity events.

**Methods:**

The Formosa Fun Coast Dust Explosion (FFCDE) was studied. Data collection consisted of in-depth interviews with clinicians from four initial receiving hospitals and their relevant hospital records. A detailed timeline of patient flow and emergency department (ED) workload changes of individual hospitals were examined to build the EDs' overload patterns. Data analysis of the multiple hospitals' responses involved chronological process-tracing analysis, synthesis, and comparison analysis in developing an integrated adaptations framework.

**Results:**

A four-level ED overload pattern was constructed. It provided a synthesis of specifics on patient load changes and the process by which hospitals' surge capacity was overwhelmed over time. Correspondingly, an integrated 19 adaptations framework presenting holistic interrelations between adaptations was developed. Hospitals can utilize the overload patterns and overload metrics to design new scenarios with diverse demands for surge capacity. The framework can serve as an auxiliary tool for directive planning and cross-check to address the insufficiencies of preparedness plans.

**Conclusions:**

The study examined a wide-range spectrum of emergency care responses to the FFCDE. It indicated that solely depending on policies or guidelines for preparedness plans did not contribute real readiness to MCIs. Hospitals can use the study's findings and proposal to rethink preparedness planning for the future beyond surge capacity events.

**Supplementary Information:**

The online version contains supplementary material available at 10.1186/s13017-021-00403-x.

## Introduction

Hospitals provide essential emergency care during patient surge following large-scale burn disasters. Most health care systems are sufficiently prepared and have enough surge capacity to respond effectively to conventional multiple casualty incidents, such as bus accidents, fire. However, less progress has been made in preparedness for large-scale events such as massive explosions.

The Formosa Fun Coast Dust Explosion (FFCDE) occurred on June 27, 2015 at the Color Play Asia water park party in New Taipei City, Taiwan. A flammable cornstarch explosion caused injuries to 499 people, and most of these injuries constituted serious burns. The average age of injured individuals was 23 years. The flammable properties of the swimwear in which attendants were dressed resulted in large total body surface area burns (TBSA, average 44%; 281 people with TBSA > 40%, 41 people > 80%) [1]. One bus and 144 regular ambulances were deployed to the field. A total of 301 (60.3%) patients were distributed to hospitals via ambulance; others were self-transported to hospitals. Within 6 h, 499 burn victims had been transported to 36 hospitals including 10 large medical centers, 23 regional hospitals, and 3 district hospitals across regions [1, 2]. These hospitals differed in their response capacity in terms of critical care bed numbers, burn care capability, accreditation level, and distance to the disaster scene. Despite the extreme patient surge and limited resources, the overall mortality rate was 3% (15 out of 499). This result was remarkable compared with international incidents [3, 4], which was acknowledged by the international emergency medicine community.

The FFCDE led to patient surge at 36 initial receiving hospitals, with the patient load exceeding several hospitals' regularly prepared surge capacity. Some hospitals experienced severe difficulties due to insufficient surge capacity in the aftermath of the FFCDE. According to the findings of the FFCDE studies [5–8], the hospitals' emergency response plans did not fully support emergency medicine in the events. The hospitals relied on adaptive responses to deal with the patient surge to generate adequate emergency care resources accordingly. These adaptations were either irregular responses or ad hoc efforts to extend medical care for burn care in ED or wards, such as adjusted bure care treatment, abnormal material mobilization, and space reconfiguration. These adaptations differed from the planned or exercised emergency care activates but were the keys to successfully dealing with the events.

There were two concerns about the FFCDE studies. Given that the emergency response plans did not properly support emergency medicine to respond to the beyond surge capacity event, how do we organize the previous papers' analyses to strengthen future preparedness planning in this circumstance? In addition, we knew that the adaptive response was mainly ad hoc in the cases studied. Simply adopting individual ad hoc responses to prepare for large-scale disasters is not a systematic approach. How can emergency response plans support emergency medicine to be ready to respond to beyond surge capacity events? Literature suggested that building a systemic model to envisioning new methods for resilient preparedness planning for disaster emergency medicine, including disaster response: anticipation, monitoring, response, recovery, learning, and self-monitoring, should be helpful [9].

The FFCDE event challenged multiple hospitals in varied demanding situations. Previous FFCDE studies revealed basic but various ways hospitals adapted to handle beyond surge capacity events. Utilizing the results of the FFCDE studies to resolve the above concerns was our motivation. The study thus aims to propose an approach of assisting hospitals in successfully preparedness planning to respond to ramifications from varying beyond-surge-capacity events. The specific objectives were to:

- 1. Synthesize patient workload data of initial receiving hospitals in emergency departments into overload patterns.
- 2. Develop an integrated adaptation framework representing hospitals’ holistic disaster responses to varied beyond-surge-capacity challenges.
- 3. Propose how to apply the present findings to assist hospitals in rethinking preparedness planning.

## Methods

### Study design

This study is part of a larger project centered on the development of resilient disaster response strategies. The study focused on analyzing the hospitals' responses to the situations they were challenged in the emergency department (ED) period, starting when the hospital was first informed of the FFCDE and ending with the discharge or transfer of all FFCDE patients from EDs. To construct overload patterns, we needed to understand better the context of the initial receiving hospitals and how their ED situations changed dynamically during the incidents. Thus, besides data associated with the influx of burn patients in the initial receiving hospitals, we collected hospitals' routine ED overcrowding information and normal capacity to facilitate a clear background and precise sequence of events for an individual hospital. To develop a holistic functional adaptation framework, we collected data associated with hospitals' responses to the MCIs through in-depth individual interviews and analysis of previously published papers related to the FFCDE, followed by chronological process-tracing analysis and comparative analysis. The study was undertaken between January 2017 and December 2018.

### Data collection

First, the study invited the initial receiving hospitals that experienced varied difficulties in responding to the aftermath of the FFCDE in order to find a wide-range spectrum of emergency care responses to the beyond surge capacity event. Specifically, based on Table 1, those hospitals that received a relatively large number of burn patients in contrast to their prior ED capacity and routine overcrowding rate were invited for an interview. Four hospitals consented to have the in-depth interviews allowing us to obtain insights into the hospitals' response to their EDs period. Additionally, the study investigated the four hospitals' critical care bed numbers, burn care capability, distance to the disaster scene, first burn patients' arrival time, etc., as shown in Table 2.

**Table 1:** Number of received casualties and approximate regular emergency department overcrowding status at the 32 initial receiving hospitals (sorted by received number of casualties)

Hospital	NoC	Number of ED beds	LOS > 48 h in ED (%)	Hospital	NoC	Number of ED beds	LOS > 48 h in ED (%)
**(MC-MM)**	55	28	7.53	RH-18	12	12	0.09
MC-H4	48	45	0.94	RH-19	9	12	0.09
MC-H1	47	160	10.57	RH-20	9	15	0.25
MC-H3	43	57	4.83	RH-9	6	33	0.22
MC-H9	32	39	6.96	RH-12	6	14	0.08
MC-H6	31	40	13.16	RH-13	6	15	7.80
**(RH-TH)**	30	20	0.08	RH-23	6	10	0.25
MC-H2	21	120	27.49	RH-8	5	30	1.90
MC-H5	20	42	14.76	RH-4	4	25	6.26
RH-22	18	10	0.25	RH-21	4	12	0.25
RH-5	15	25	0.01	RH-6	2	20	0.06
RH-10	15	31	0.74	RH-7	2	27	0.09
MC-H7	14	26	7.53	RH-14	2	15	0.10
**(MC-WH)**	14	26	0.28	RH-16	2	13	0.80
RH-2	13	40	1.73	RH-1	1	30	6.46
**(RH-SH)**	13	34	2.56	RH-15	1	15	1.15

Data source: National Health Insurance Administration, open data on health care quality. https://www.nhi.gov.tw/AmountInfoWeb/search.aspx?Q5C1_ID=2&Q5C2_ID=1652

NoC, number of received casualties; ED, emergency department; LOS, length of ED stay; MC, medical center; RH, regional hospital; MM, Mackay Memorial Hospital Tamsui Branch; SH, Shuang Ho Hospital; TH, Taipei Hospital, Ministry of Health and Welfare; WH, Taipei Municipal Wan Fang Hospital

The interviewed hospitals are presented in parentheses

**Table 2:** Capacity and workload characteristics of the four interviewed hospitals

		MM(1)	TH (2)	SH(3)	WH(4)
	Public/Private	Private	Public	Private	Private
	Jurisdiction	NTC	NTC	NTC	TC
	Accreditation level	MC	RH	RH	MC
	Emergency responsible hospital	Severe level	General level	Severe level	Severe level
Capacity	Total number of beds	1009	517	1130	726
	Total number of burn beds	0	0	0	4
	Hospital staffing	1723	751	2206	1667
	ED staffing/day^@^	38	20	39	40
	Number of plastic surgeons	4 AP + 4 RP	1 AP^**^	2 AP^*1^	6 AP
	Number of ICU beds/average occupancy rate	53/ 95%	30/ 90%	72/ 92%	47/ 94%
	ED capacity (observation beds + resuscitation beds)	28 + 4	20 + 3	34 + 4	26 + 4
	Driving time from the disaster scene	~ 20 min	~ 22 min	~ 27 min	~ 47 min
	Arrival time of the first burn patient (disaster time 20:32)	21:07 (0.5 h)	22:04 (1.5 h)	22:17 (1.6 h)	23:35 (3 h)
	Received/registered FFCDE patients	60/44	30/29	13/11	15/14
Workload	Burn severity (TBSA [average])*	1–90%, (48.6%)	5–72%, (36.3%)	10–85%, (51.3%)	8–70%, (40.6%)
	Intubated patents in ED	20	0	4	4
	Number of ED patients before the FFCDE patients’ arrival	43	17	27	26
	Number of non-FFCDE patients admitted during MCI	13	36	45	25

MM, Mackay Memorial Hospital Tamsui Branch; SH, Shuang Ho Hospital;

TH, Taipei Hospital, Ministry of Health and Welfare; WH, Taipei Municipal Wan Fang Hospital; NTC, New Taipei City; TC: Taipei City; AP, attending physician; RP, resident physician, TBSA, total body surface area; MC, medical center; RH, regional hospital, EM, entrusting management

^*^ Only registered burn patients were included in the analysis because the data were incomplete

^**^This AP did not present on the FFCDE night

^@^ Total number of clinicians in 2 shifts for physician and 3 shifts for nurse per day

Table 2 presents details on the characteristics of the four interviewed hospitals. Mackay Memorial Hospital Tamsui Branch (hereafter Mackay Memorial (MM) Hospital), which was the closest in proximity to the FFCDE scene among all the four hospitals, received the highest number of burn patients (60) among all hospitals on the night of the disaster. It also received patients earlier than any other hospital. Taipei Hospital (TH), Ministry of Health and Welfare, the smallest of the four hospitals, received the largest number of burn patients (30) among regional hospitals. Despite being large hospitals, Shuang Ho (SH) Hospital and Taipei Municipal Wan Fang Hospital (WF) received fewer burn patients than MM Hospital or TH Hospital because they were farther from the disaster scene.

Each hospital's response was examined using the critical incident technique [10, 11]. First, hospital records were reviewed, including ED admission logs specifying patient arrival times, departure types (discharge or transfer), departure times, patient type (FFCDE or non-FFCDE), and patient triage level. These data built a detailed timeline of patient flow and ED workload changes of individual hospitals. Next, the researchers conducted extensive, in-depth, semi-structured interviews with 34 participants across multiple levels of each of the four hospitals. The content of the interviews is described in more detail in individual publications regarding two of the hospitals [6, 7]. Finally, the data were corroborated with detailed patient information from hospital records.

### Data analysis

The data analysis sequentially progressed through several stages hospital by hospital. First, the process tracing method [12, 13] was used to organize the data collected through the critical incident technique for each hospital by time and process functions. ED’s workload was measured by three indicators: load accumulated index (LAI), load relief index (LRI), and ED overload time to compare the relative difficulties across hospitals. LAI was calculated as (cumulative maximum number of patients in ED—number of ED patients before the FFCDE patients’ arrival) / number of 15-min intervals in the workload ascent period. LRI was calculated as (cumulative maximum number of patients—total FFCDE and non-FFCDE patients in the turning point resuming regular ED work) / number of 15-min intervals in the resuming period. Overload time is how long the ED’s workload is above normal ED capacity.

Next, the study started from hospital TH to thematically synthesize specific responses of practitioners and deployed them into categories of functional adaptations. The functional adaptations were then plotted to generate an integrated adaptation framework to allow the analysis of interactions among individuals, small groups, units, hospitals, and external organizations. This framework became a structural platform upon which the hospitals' subsequent comparative analysis was based. And it was revised when a new functional adaptation was found in other hospitals' analyses.

## Results

### Identification of patient overload patterns in ED period

**Fig. 1:**
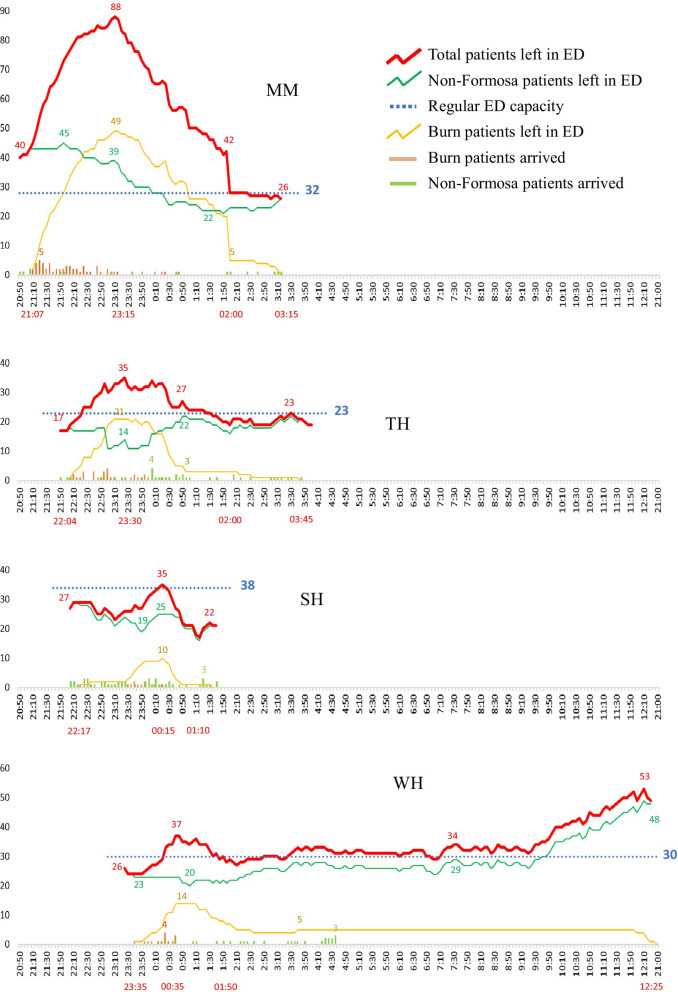
Patient surge and workload patterns

The FFCDE caused some of the initial receiving hospitals to exceed their regular surge capacity. The overload in each hospital had three dimensions: number of burn patients, number of acute patients, and pattern of patient arrival. Based on Table 2 and ED admission logs, four patterns of hospital workload change over time were identified chronologically. MM Hospital is the closest emergency responsible hospital (ERH) to the disaster scene. Taipei Hospital, a public ERH, is located at a 22-min drive from the disaster scene. Therefore, a sudden influx of burn patients transported in ambulances or private cars quickly overwhelmed their systems. Figure 1 illustrates the timeline at each hospital of patient surge and workload, from the first burn patient's receipt to the discharge or transfer of all FFCDE patients from the ED.

The FFCDE occurred at 20:32. The study marked 20:50 as the beginning of the x-axis. From this point on, the four hospitals show four types of dynamic overload patterns in Fig. 1. Each hospital's LAI, LRI, and overload time are shown in Table 3. The study used only LAI and overload time to determine a specific difficulty level for individual hospitals. LRI was biased due to hospitals' different adaptation strategies of serving non-FFCDE patients; see the number of non-FFCDE patients admitted by each hospital during the mass casualty incident (MCI) in Table 2. Based on the value of LAI and overload time: "the higher LAI, the worse the situation; the longer overload time, the worse the situation," each hospital was assigned a difficulty level from the four codes: extreme, high, moderate, and low to represent their workload pattern (Table 3), e.g., hospital MM was recognized as in the extreme level of difficulty.

**Table 3:** Patients workload change in emergency department

Workload	MM Extreme	TH High	SH Low	WH Moderate
a. 1st burn patient arrival time	21:07	22:04	22:17	23:35
b. Number of ED patients before the FFCDE patients’ arrival	43	17	27	26
c. Cumulative maximum number of patients or turning point of patient decline	88	35	35	37
d. Time in point c	23:15	23:30	00:15	00:35
e. Number of 15-min intervals in the ascent period (e = (d-a)/15)	9	6	6	4
f. Time in resuming regular ED work	02:00	02:00	01:10	01:35
g. Total FFCDE and non-FFCDE patients in point f	28	19	22	28
h. Number of 15-min intervals during the ED resuming period (h = (f-d)/15)	11	10	4	4
Overload time (hour): workload above regular ED capacity (blue line)	21:07–02:00 (5)^**+**^	22:20 -01:20 (3)	0:10 – 0:30 (0.5)	0:20 – 1:50 (1.5)
Load accumulated index(LAI) (c-b)/e	5	3	1.33	2.75
Load relief index(LRI) (c-g)/h	5.45	1.6	3.25	2.25

^+^ MM hospital had already exceeded its regular ED capacity when the mass casualty incident occurred. We used the arrival time of the first FFCDE patient as the beginning overload point

### A generalized integrated framework of functional adaptations

Chronological process tracing analysis focused on how the ED and other units adapted to cope with the difficulties created by the patient surge in and out of EDs. The study identified 19 functional adaptions across hospitals. A detailed description of the 19 adaptations is in the supplemental material (Additional file 1). Taipei Hospital was the first one to undertake this analysis. The analysis identified significant adaptive responses and their interconnections across these actions and then developed an integrated functional adaptation framework with 14 response categories. Through the analysis of the other three interviewed hospitals’ responses by using this framework as a basis, we identified two new dimensions of functional actions performed by the MM hospital: reorganization and reordering of ED (Function 10: F10) and transfer of burn patients to other hospitals (F14) from the ED. In addition, the two adaptations (F0-1 and F0-2) observed across all the hospitals before or after the arrival of the first burn patient were incorporated into the framework. Thus, an enriched framework was built upon the generalized 18 functional adaptations developed in response to the MCIs of the four interviewed hospitals.

**Fig. 2:**
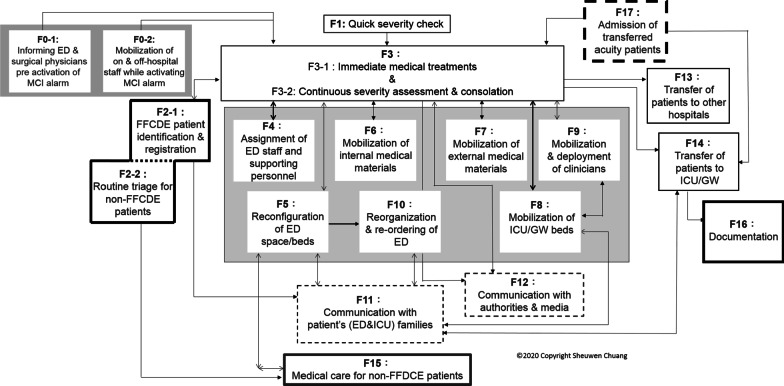
An integrated functional adaptations framework in response to mass-casualty incidents after the FFCDE

Beyond the 18 functional adaptations, the study found a unique emergency adaptation in the additional two medical centers through literature review [8, 14]. During their emergency responses to the ED period, in addition to receiving victims from the disaster scene, the two medical centers received burn patients transferred from low-level hospitals and admitted them to EDs or, unconventionally, directly to the ICU. Thus, an extra adaptation was incorporated into the framework: admission of transferred acute patients (F17). The final framework (Fig. 2) comprised 19 functional adaptations resulting from the receiving hospitals' initiatives taken and adjustments made to the regular protocols to expand emergency care capabilities, including hospitals' response to varying scales of MCIs, ranging from extreme to low difficulty. The interconnecting lines among the adaptations show the interrelationships and interdependence between the adaptations.

### Comparison of adaptations in beyond-surge-capacity situations

Besides developing the overall generalized adaptation framework and the four overload patterns that exerted varying levels of stress on the hospitals, the study compared the hospitals' notable unique situations and their corresponding adaptations among the four hospitals (Table 4). The hospital with extreme difficulties (MM hospital) had the slightest information regarding the MCI but the most rapid influx and highest number of burn patients. The hospital did not stabilize to manage the mass casualties until the challenges regarding the emergency care staff and ED space were resolved, approximately 50 min after the emergency response program alarm sounded. In the case of the high-level difficulty hospital (TH), they had no burn care capacity. Their adaptations to the MCI involved anticipatory actions, early mobilization of the surgical team to deal with the shortage of burn specialties, and the implementation of care flexibly under the control of the surgical department.

**Table 4:** Notable situations and adaptations between overload patterns in the mass-casualty incident

Difficulty level	Notable unique situations	Notable unique adaptations
Extreme (Mackay Memorial Hospital Tamsui Branch)	No clear news had been reported regarding the disaster when the first seven burn patients were received The burn patients arrived unexpectedly quickly Several acuity patients required emergency intubation, but intubation experts were not immediately fully available The already overcrowded ED became swiftly congested with burn patients, on-and-off-duty staff members, and non-hospital persons The workload peak was almost four times the normal capacity Shortage of space, bag valve masks, portable ventilator equipment, stretchers, and ambulances	**(F0-2)** ERP alarm was activated to call for assistance from onsite staff members after two severe burn patients were treated **(F4)** An ED physician urgently called for job prioritization of on-duty physicians to assist with intubation. In total, 20 patients were intubated in the ED **(F3-1)** Simple treatments for low-acuity patients (e.g., wound flushing and covering with wet gauze) were administered at the triage area before the patients were moved into the main ED. Only 10 patients were treated with burn ointment in the ED **(F10)** An ED manager of the MM network hospital initiated the reconfiguration of the chaos ED space upon his arrival 50 min after that of the first wave of burn patients **(F5)** Irregular rooms (e.g., meeting rooms, and shower rooms) were opened to allow low-acuity patients to flush their own burns to reduce wound temperature and pain **(F7)** A young non-ED physician who anticipated that burn ointment would run out after patient transfers requested an urgent delivery of burn ointment and other supplies from vendors
High (Taipei Hospital, Ministry of Health and Welfare)	The hospital VP saw an early-stage news report offsite regarding the disaster The hospital has no burn specialists and little experience with burn care The director of the surgical department took charge of the emergency care of burn patients The hospital had no prior experience with MCIs involving more than 15 patients Shortage of burn care supplies	**(F0-1)** Anticipating an influx of burn patients and a shortage of burn experts and clinicians, the VP proactively initiated an alerting call to mobilize ED and surgical managers **(F0-2)** The ERP alarm was activated to call for assistance from onsite staff members before the burn patients’ arrival **(F3-1)** No acuity patients were intubated in the ED, but one was intubated in the ICU. All patients were treated with burn ointment in the ED **(F6)** A nurse returned to her ICU for more burn ointment **(F7)** External supplies were mobilized from alliance hospitals to the ED in the early stages of the MCI
Moderate (Taipei Municipal Wan Fang Hospital)	This hospital was informed that it would be receiving a large number of patients with mild burns transported by bus. But, the actual number was lower and distributed by ambulances, some of the patients had severe burns No beds were available in the burn ward or the ICU	**(F5)** The hospital lobby adjacent to the ED was prepared as a temporary treatment area waiting for all patients with mild burns. They were later moved back to the ED **(F3-1)** The acuity patients were given preliminary treatment in ED. Three of them were intubated in the ED and then quickly transferred to the ICU or the burn ward after relocation of the existing patients in ICU and burn ward
Low (Shuang Ho Hospital)	The hospital was informed of the incoming arrival of lots of burn patients The available ED space was larger than that in the other hospitals The burn patients’ arrival was slow. And the ED workload was below regular ED capacity in this night	**(F0-2)** The ERP alarm was activated to call for assistance from onsite staff members after two burn patients had already arrived (F5) To streamline patient flow for triage and registration, the ED was divided into two areas with different routes for FFCDE patients and non-FFCDE patients **(F3-1)** An otolaryngologist used an endoscope to check the patients’ respiratory tracts

ERP, emergency response plan; VP, vice president of the hospital; ED, emergency department;

ICU, intensive care unit; MCI, mass-casualty incident; FFCDE, Formosa Fun Coast Dust Explosion

The moderate-level hospital (WF) had inconsistent information on the incoming mass casualties that differed before and after the arrival of burn patients. Patients with mild burns were treated in the prepared hospital lobby (adjacent to the ED) to make room in case of the arrival of more burn patients, and the high acuity patients were transferred to the burn ward as soon as possible. The preparation of ED space was not fully utilized afterward because the hospital received fewer burn patients than the numbers informed by the Emergency Operation Center earlier. The low-level difficulty hospital (SH) had the largest ED space and patient care capacity of all the hospitals. So this allowed the hospital to comply with some of the standard procedures of emergency medicine, such as using the MCI numbering system for burn patient registration and medication prescriptions. And SH resumed regular ED work quickly.

The analysis revealed that substantial variations in organizational characteristics—the lack or absence of burn specialists, shortage of medical supplies, congestion, and insufficient staffing in the ED— led the hospitals to adopt different adaptational strategies and generate various cascade effects accordingly. Table 5 compares major adaptations implemented by the hospitals and typical cascade effects according to contextual situations. All the hospitals with insufficient staffing capacity adopted common strategies to maximize their efficiency (i.e., F4, F9, F13, F14, F15, F16, and F17). However, all hospitals did not avoid the duplication or lack of complete patient identification, and the list of mass casualties was incomplete and imprecise.

**Table 5:** Comparison of major adaptations between contextual situations in the mass-casualty incidents

Context	Notable typical adaptation examples	Cascade effects
***Number of burn specialists***: None-burn specialist /plastic surgeon hospital (C1) versus A few-burn specialists hospital (C2)	Unique to C1: **F0-1**: Anticipation and proactively initiated to mobilize ED and surgical managers before receiving EOC’s call **F1**: Triage was performed by triage nurses and ED physicians **F3-1**: Burn dressing in ED and intubation in ICU Unique to C2: **F1**: Triage by ED physicians and plastic surgeons or burn specialists **F3-1**: Intubation in ED and burn dressing in wards Common to C1 & C2: **F3-1**: Team structure was flexible, and nurses were trained in time on burn care in the ED **F14**: Simplified transfer of burn patients to other hospitals from ED or wards	C1: Approaching run out burn ointment C2: Approaching the gridlock of ED space, and quick shortage of intubation devices and equipment
***Congested ED space*** Farer hospital continuously receiving burn patients with uncertainty (C3) versus Closest hospital continuously receiving mass casualties with uncertainty (C4)	Unique to C3: **F5:** Three types of mass relocation: 1) Initial relocation of few non-FFCDE patients inside ED, followed by mass relocating all non-FFCDE patients into hospital lobby; 2) one-time mass relocation of non-FFCDE patients into a separated area from victims in ED; 3) located all mild-injured burn patients in hospital lobby adjacent to ED, moved them all back to ED afterward **F2-1**: Use of mass casualty registration numbering protocol from the first burn patient Unique to C4: **F5**: Stepwise relocation—ED physicians kept looking space and relocating patients in ED until gridlock was noticed **F2**-1: First, use of emergency registration procedure, then switched to MCI registration numbering protocol **F3-1**: Minor injured were first treated in triage area outside ED, allow low-acuity patients to flush their own burns, deferred coating **F10**: Reconfigured patients’ layout and asked to hang a visible ID paper of burn patients to the drip stand of each bed, moved out the chairs in waiting room, and strictly asked ED access control	C3: Mitigated getting overcrowded ED C4: Congested ED space impacted on F3-1 Deferred coating ointment led the shortage of burn ointment later Recovered ED order made patients transfer smoothly Duplicate or missed patient IDs were created
***Shortage of medical materials*** Materials shortage in ED (C5) versus Materials shortage in wards (C6)	Unique to C5: **F6**: Mobilization and deployment of internal materials bypassed standard inventory procedures including admission of control drug, borrowing burn materials, tubes, Ambu, and portable ventilator equipment from other units. borrowed stretchers from 119 ambulances, requested for ambulances by formal and private channels Unique to C6: **F7:** A top manager and a young physician anticipated run out of medical materials for burn care to proactively mobilized external materials	C5: Deferred coating in ED Arranged burn patients to sit in chairs and waiting for stretchers C6: No shortage of burn materials in wards
***Insufficient staff for burn care, registration and documentation in ED*** ***(***Common situation across hospitals)	**Common adaptation: maximization of staff capacity** **F4**: Use of flexible team structure, nurses assisted the triage for non-burn patients and registration, staff worked overtime to extend care from ED to wards. senior physicians volunteered to escort the transferred patients in ambulances **F9**: Multiple channels (formal and informal) to call for offsite staff, senior managers called the EOC to stop sending **F13, F14, F17**: Simplified transfer procedures through oral handoff, plus limited documents provided afterword **F15**: Discharged relatively lower risk non-FFCDE patients **F16**: Medical documentation was deferred and simplified, reporting mass causally list to MOHW on line was deferred	Faster treatment Duplicate or missed patient IDs were created Incomplete medical documentation Incomplete and imprecise mass casualties list in time

ICU, intensive care unit; MOHW, Ministry of Health and Welfare; EOC, emergency operation center

Regarding the mitigation of congested ED space, based on ED size and bed numbers and the pattern of the influx of mass casualties, the hospitals reconfigured their ED space through two relocation approaches: stepwise and mass relocation. Stepwise relocation involved reactively moving a few non-FFCDE patients to increase capacity for burn patients' influx gradually. This approach was used by Mackay Memorial Hospital, which, as mentioned, was the closest to the disaster scene, that faced the most difficult situation and had the least awareness of the disaster at the beginning of the FFCDE. The cascade effect of quickly congested ED affected F3-1 and triggered F10 to resume order in the ED. By contrast, the other hospitals proactively conducted mass relocation through three methods according to their available ED space and the warning information from the Emergency Operation Center.

## Discussion

Hospital staff are typically required to follow formal protocols, i.e., escalation policies or legal/regulatory policy, or emergency response programs, to respond when demand increases or capacity is reduced in emergency events [15, 16]. However, these policies are distillations that fail to capture real difficulties faced by practitioners, lack of illustration about event dynamics, or the full extent of the early adaptations of initial receiving hospitals. The study evidenced that only depending on the policies or guidelines for the preparedness plan did not contribute readiness to the varied scales of MCIs altogether. To achieve the study’s objectives, the paper highlights the following themes: preparation for varying surge capacity, preparing system capacity, and adapting to longer sustained time, to support learning and rethinking preparedness planning for beyond surge capacity events.

### Surge capacity preparation

Surge capacity is generally defined as an ability to evaluate and care for a substantially increased volume of patients that exceeds normal operating capacity. Specifically, hospitals with different levels of surge capacity following a mass casualty incident fall into three basic categories depending on the magnitude of the event to individual hospitals: conventional, contingency, and crisis. Conventional capacity was defined as “The spaces, staff, and supplies used are consistent with daily practices within the institution.” Contingency capacity was defined as “The spaces, staff, and supplies used are not consistent with daily practices but maintain or have minimal impact on usual patient care practices.” Crisis capacity was defined as “Adaptive spaces, staff, and supplies are not consistent with usual standards of care but provide sufficiency of care in the setting of a catastrophic disaster (i.e., provide the best possible care to patients given the circumstances and resources available)” [17].

During the MCIs after the explosion, the initial receiving hospitals were pushed into a position that required them to develop additional surge capacity for the provision of emergency care to the mass casualties. The present study assigned each of the four hospitals a difficulty level (extreme, high, moderate, and low) of responding to the MCIs. According to the definitions of the three basic categories of surge capacity, "extreme" can be implied as a crisis circumstance for the surge capacity, "high" and "moderate" indicated as a contingency, "low" suggested as a conventional situation. And Tables 4 and 5 showed how individual hospitals adaptively responded to the difficulties in each level, based on their contextual concerns. The study indicated that hospitals might face the demanding challenges of any of the three categories of surge capacity after an unexpected beyond-surge-capacity event. Hospitals should be better-prepared staff to offer patients timely and appropriate care, no matter their accreditation level. Referring to the study’s synthesized findings, f hospitals can learn how to expand their disaster preparation investments in multiple simulation cases for surge capacity planning from this one case. They can use Fig. 1 (overload patterns) and Tables 3, 4 and 5 for the planning to capture an overall understanding of what challenges could occur over time and how hospitals responded to these difficulties to extend emergency care in each category. Also, emergency planners can consult the responses described in more detail in individual publications regarding two hospitals at an extreme and high level [6, 7].

### Using the integrated adaptation framework to support system capacity planning

The FFCDE event challenged multiple hospitals in varied demand for surge capacity. The initial receiving hospitals adaptively provided the best possible care to the patients based on their available personnel, equipment, and supplies. The beyond surge capacity event analysis revealed several primary responses to approaching or reaching saturation (overload & shortages) and revealed essential functions for successful adaptation (Table 4 and 5 and reference 6, 7). In terms of four critical interdependent factors (four Ss) that contribute to an effective surge response: system, space or structure, staff, and stuff (i.e., supplies and equipment) [17, 18], the study synthesized hospitals' adaptations into 19 functional adaptations for the burn MCIs. Each adaptation involves specific goals for expanding capacity, e.g., clinicians, ICU beds, medical materials, according to the characteristics and contextual situations of the particular hospitals. In addition, the study findings indicated that the adaptations to mobilize and deploy resources must root in good coordination and communications across units and functions to be successful.

Emergency experts advocated that the other three variables cannot be appropriately managed without the underlying system components, although each of these "four Ss" is important to responses to MCIs [18]. And, in preparedness planning for overwhelming MCIs, having a capable "system" capacity is imperative for seamless integration with the other three capacities for the varying scale of incidents. System capacity generally refers to integrated policies and procedures (e.g., the 4Cs: command, communication, coordination, and control) for effective disaster response management [16]. The links between individual adaptations in the integrated 19 functional adaptations framework present the coordinated interactions, communication, and interdependency (command and control) across units and functions to cope with specific situations accordingly. The integrated framework reflects a holistic and systematic structure of the development of the four Ss in response to the MCIs and provides practical knowledge for preparedness planning. The framework itself demonstrates "system" capacity that shows the interrelations among adaptations via the 4Cs, which can help emergency planners make projections regarding the four Ss' systemic cascade effect.

Successful responses to MCIs are wholly dependent on effective coordination and communication between individuals and across units and roles during critical stages [19]. By examining the adaptation framework and the interrelations between adaptations, hospitals can obtain a systemic understanding of holistic disaster response and interaction needs, which they can use the integrated knowledge to redesign existing preparedness plans to support coordinated cross-unit adaptations. Besides, the evaluation of results from ongoing planning plays a vital role in preparedness planning [20]. The present framework can then serve as a visualizable checking tool to direct practitioners’ attention. Emergency planners can address or mark the weaknesses of system capacity on the framework when evaluating, organizing, practicing, and implementing preparedness plans for a specific scenario [20].

### A complementary approach adapting to longer sustained time

The intensified pressure and the increased scale of demand caused by the FFCDE far exceeded the hospitals' reasonable expectations or planning capacity. Examining the varying adaptations presents a specific opportunity to learn regarding the most effective response to the same event. The FFCDE event and the study indicated that preparatory investments are needed to have the adaptive capacity when challenges arise in the future. The four overload patterns and corresponding metrics show four types of patient surge arriving hospitals and the varied overload time before resuming to normal operations in EDs. Notably, the overload caused by the influx of FFCDE patients was shorter in duration than that in other large-scale events, such as an earthquake crisis. If the MCI had extended for a longer period, strain from overwork and sustaining adaptations might have led to clinician attrition. To invest in building adaptive capacity in advance for such beyond-surge capacity events need to be considered.

This paper suggests using the four overload patterns with the overload metrics and the integrated adaptation framework as a complementary approach to supporting preparedness planning for beyond surge capacity events. The four overload patterns provide varying situations that more comprehensively illustrate patient load changes to EDs against the prepared surge capacity over time than conventional MCI guidance. Suppose emergency planners consider additional scenarios associated with longer overload time. They can adapt the approach to develop new scenarios with longer overload time and indicate the gaps between capacity and loading in each simulation. Subsequently, the integrated adaptation framework can be used as a directive map guide to compare the current plan and identify planning insufficiencies. Doing so would facilitate understanding differences in hospitals' overload situations, the cascade effects of possible adaptations, and preparedness planning improvement in advance.

## Conclusions

Large-scale mass casualty incidents that overwhelm health care systems have become normal in recent decades. The development of effective preparedness plans that can deal with varied beyond-surge-capacity events is an essential action generating real readiness for future disasters. Hospitals without actual burn MCI experience may have less confidence in their preparedness and may be more motivated to improve readiness by following itemized benchmarks [20]. The study examined a wide-range spectrum of emergency care responses to a beyond surge capacity event and found that most of the 19 adaptations and the coordination resulted from individual initiatives and adjustments to regular protocols rather than an inculcated and practiced MCI preparedness plan. Therefore, solely depending on the policies or guidelines for the preparedness plan did not contribute readiness to the varied scales of MCIs. The proposed complementary approach to support preparedness planning includes a four-overload pattern illustrating the diverse situations of MCIs after a beyond surge capacity event and an integrated adaptation framework presenting a holistic perspective for the 4Ss’ surge capacity planning. This approach can help hospitals rethink how to produce better plans that actually generate real readiness to respond to a beyond surge capacity event. But we haven’t laid this out sufficiently in this paper, only the beginning. Future work would apply this approach in real systems and then examine how it works when a crisis strikes.

## Supplementary Information

**Additional file 1.** Description of the 19 functional adaptations.

## Data Availability

An additional file introducing the 19 functions adaptations is included in the Additional file 1

## Abbreviations

4Cs: Command, communication, coordination, and control
ED: Emergency department
ERH: Emergency responsible hospital
FFCDE: Formosa Fun Coast Dust Explosion
ICU: Intensive care units
LAI: Load accumulated index
LRI: Load relief index
MCI: Mass casualty incidents
MM Hospital: Mackay Memorial Hospital Tamsui Branch
SH Hospital: Shuang Ho Hospital
TBSA: Total body surface area burns
TH: Taipei Hospital
WF: Taipei Municipal Wan Fang Hospital
