# Supplementary material for: Rethinking preparedness planning in disaster emergency care: lessons from a beyond-surge-capacity event
Source: World J Emerg Surg. 2021 Nov 29;16:59. doi: 10.1186/s13017-021-00403-x (PMC8628445; doi:10.1186/s13017-021-00403-x)
Supplement: Supplementary file 1 — Additional file 1. Description of the 19 functional adaptations. [file 13017_2021_403_MOESM1_ESM.docx]

**The 19 functional adaptations**

1. Medical care:
2. Emergency medical treatment for burn patients including first aid, resuscitation, and continued assessment and consolation (F1, F3-1, F3-2)

F1) Quick severity checks that shorten the triage process to allow prompt assignment of patients into groups of similar care requirements were performed differently in the initial hospitals. Also, severe patient triage was bypassed before activating the MCI alarm in the hospitals that received casualties in the very early stages.

F3-1) To prevent patient deterioration, standard immediate first aid for burn victims (e.g., resuscitation, vital sign assessment, airway checks, intubation, early wound cleaning, dressing, and admitting control drug) was administered in varying extents, according to the resources available. This was performed using a flexible team structure as more patients and staff members arrived.

F3-2) Continuous severity assessment involving patient status monitoring and consolation to relieve both their physical and mental pain and distress while they waited to be transferred. This was performed by a nurse, a flexible team, or medical volunteers according to the hospital context.

1. Burn patient transfer (F13, F14, F17)

F13) Simplified transfer procedures for burn patients to ICUs or general wards within the same hospital. The procedures were conducted through oral handoffs, with limited documents provided and secondary triage afterward.

F14) Simplified procedures for the transfer of high-acuity burn patients to the ICUs of intervention-capable hospitals or low-acuity burn patients to hospitals near their residences following their families’ requests.

F17) Simplified transfer procedures for receiving high-acuity burn patients from other low-level hospitals to EDs or ICUs in medical centers.

1. Rearrangement of regular emergency care services (F2-1, F2-2, F15, F16)

F2-1) Adjusted FFCDE patient identification and registration were conducted by nurses to allow immediate treatment to proceed smoothly.

F2-2) Routine triage for new non-FFDCE patients was performed by the hospitals to varying extents. For those patients triaged to low acuity classifications, they were informed of a prolonged wait and had the option to seek care elsewhere voluntarily

F15) Discharged relatively low-risk non-FFDCE patients from the ED.

F16) Deferred or simplified medical documentation. Registration workflow was mixed, and nurses were involved to support patient administration i.e. patient registration.

1. Logistic support
2. Mobilization and reconfiguration of relevant resources (F0-1, F0-2, and F4 – F10**)**

F0-1) ED and surgical physicians were informed of the FFCDE by high-level management upon the release of news reports, which was prior to the arrival of patients. This initiative triggered the early mobilization of physicians before MCI confirmation.

F0-2) The MCI alarm was activated and distributed via formal channels and the message platform LINE in private groups to mobilize on-and-off-duty staff at the beginning of an MCI.

F4) Formal task assignment and voluntary participation was undertaken to conduct the emergency medical care. Off-duty supporting personnel returned to help with emergency care, material deployment, registration, patient transfer, social work, etc.

F5) The ED space was reconfigured (through stepwise or mass patient relocation) to support immediate treatment of the influx of burn patients at the beginning of and during the MCI. ED observation beds were used for inpatient care for the low-acuity burn patients after the ED resumed normal operations.

F6) To support immediate medical treatment in the ED, internal medical resources were mobilized through multiple channels in both regular and irregular processes. Standard inventory procedures were bypassed in the admission of control drugs and the borrowing of burn care supplies, tubing, bag valve masks, and portable ventilator equipment. Stretchers were borrowed from ambulances, and private-channel requests for ambulances were made.

F7) To support immediate and follow-up medical treatment in both the ED and wards, external medical resources were mobilized through multiple channels. For example, a high-level manager or a young physician anticipated the depletion of burn care medical supplies and proactively initiated additional preparations.

F8) Some non-FFCDE patients were transferred unexpectedly from intensive care units to general wards, where a concentrated acute care area was set up for the burn patients. An ED central control physician, i.e., ED manager or General surgery manager, coordinated the distribution of ICU/general ward beds to allow for the efficient transfer of patients from EDs.

F9) Central control via multiple channels was used to mobilize and deploy clinicians for emergency care and follow-up treatment after patient transfer to ICUs or wards.

F10) After adopting the stepwise patient relocation in ED to cope with the continuous influx of patients and external supporting persons, the closest hospital faced the gridlock of ED space that significantly affected emergency care efficiency. The hospital reconfigured its ED space again to improve the entry access control, to streamline the triage and workflow efficiency of emergency treatment, such as the scattered beds were reconfigured into lines; each patient was only permitted one bedside family member; a piece of A4 white paper with FFFCDE patient name and temporary number to the drip stand of each bed was additionally attached to distinguish the FFCDE and non-FFCDE patients effectively.

1. Public communication (F11 and F12)

F11) Staff members communicated with and attempted to persuade the families of non-FFCDE patients admitted in ICU to relocate those patients in order to open beds for the severe burn patients. The information of FFCDE patients was provided through varied channels i.e. patient information written on a whiteboard in ED, an ED information help desk, to their families or friends.

F12) The hospitals communicated with authorities and the media in a timely manner; however, some hospitals declined media interviews.
